# Ink Formulation and Printing Parameters for Inkjet Printing of Two Dimensional Materials: A Mini Review

**DOI:** 10.3390/nano11123441

**Published:** 2021-12-19

**Authors:** Ho-Young Jun, Se-Jung Kim, Chang-Ho Choi

**Affiliations:** 1 Department of Chemical Engineering, Gyeongsang National University, Jinju 52828, Korea; jhy1848@gnu.ac.kr; 2 School of Chemical Engineering, Jeonbuk National University, Jeonju 54896, Korea; sejung.kim@jbnu.ac.kr; 3 Department of Materials Engineering and Convergence Technology, Gyeongsang National University, Jinju 52828, Korea

**Keywords:** 2D materials, inkjet printing, ink formulation

## Abstract

Inkjet printing of two-dimensional (2D) material has been a center of interest for wearable electronics and has become a promising platform for next-generation technologies. Despite the enormous progress made in printed 2D materials, there are still challenges in finding the optimal printing conditions involving the ink formulation and printing parameters. Adequate ink formulation and printing parameters for target 2D materials rely on empirical studies and repeated trials. Therefore, it is essential to compile promising strategies for ink formulation and printing parameters. In this context, this review discusses the optimal ink formulations to prepare stable ink and steady ink jetting and then explores the critical printing parameters for fabricating printed 2D materials of a high quality. The summary and future prospects for inkjet-printed 2D materials are also addressed.

## 1. Introduction

Since the discovery of graphene, two-dimensional (2D) layered materials have attracted great attention in various research fields due to their large surface area and unique quantum confinement effect [1]. The unique properties of 2D materials provide abundant opportunities for next-generation applications and technologies [2]. In order to turn these opportunities into the real, scalable production of 2D nanosheets (NSs), it should be accompanied by the advancement of deposition techniques. Liquid phase exfoliation (LPE) has proved its ability to produce scalable and high-yield 2D NS dispersion. When it comes to promising deposition techniques, several candidates have been introduced, including spin coating [3,4], spray coating [5,6], inkjet printing [7,8,9], and screen printing [10,11]. Among them, inkjet printing is a high-volume and low-cost manufacturing process, allowing complex and large-area patterning of 2D NSs, and has been applied to various fields such as electronics, bio, and optics based on inorganic or organic materials [12,13,14]. Based on the development of various 2D NS inks, inkjet-printed 2D NSs have been flourishing in many research fields [15,16].

Graphene, transition metal dichalcogenides (TMD), boron nitride (BN), black phosphorus (BP), and MXenes are common 2D NSs for inkjet printing [17,18,19,20,21]. These 2D NSs possess different intrinsic properties and thus correspondingly demand different ink formulation strategies [15]. The viscosity, surface tension, and concentration are critical factors to be considered in designing the ink formulation of 2D NSs [16,22]. For most cases, these factors interactively determine the ink stability and ink-jetting dynamics. Once stable droplets are discharged from nozzles, they encounter diffusion and evaporation that simultaneously occur on the substrate. The diffusion and evaporation should be tailored to accomplish uniform printing patterns [23]. Like the ink formulation, the solvent’s viscosity and surface tension play a vital role in tuning the diffusion and evaporation rates. Additionally, some operating parameters of inkjet printing also affect the quality of printed 2D NSs. Overall, the 2D NS-inkjet printing is not straightforward, and all these parameters should be considered interactively. Some review articles have recently been published, providing general information and insights ranging from ink formulation to the application of printed 2D NSs [15,16,24]. In addition to such comprehensive reviews, a mini-review article focusing on optimizing the printing conditions for both ink formulation and the printing process is also worthy of being reported. This article is composed of several sections. The first section deals with ink formulation strategies, starting with exfoliation and then moving on to formulation. The second begins with the working principles of inkjet printing and takes a significant portion to discuss the operating conditions of inkjet printing to achieve high-quality printed 2D NSs. A brief explanation of the applications of printed 2D NSs is assigned to the third one. Lastly, a summary and future respective are presented. This review will offer sound guidance for relevant researchers seeking adequate ink formulation and printing parameters for their target 2D NSs.

## 2. Ink Formulation of 2D Materials

The first step for an ink formulation of 2D materials is to exfoliate the bulk 2D materials into thin-layered 2D nanosheets (NSs). There have been many exfoliation methods designed to obtain 2D NSs with high quality, high yield, and excellent processibility for target applications [25]. Since the 2D material ink should be formulated into a colloidal dispersion, liquid-phase exfoliation (LPE) is generally used to produce 2D NSs [1]. The physical properties of 2D materials are diverse, depending on their structure and composition. This means that each 2D material needs its optimal exfoliation conditions involving an exfoliation medium, additives, and exfoliation equipment parameters [16]. Once the 2D NSs are prepared by exfoliation, the following step is to formulate the ink using the exfoliated 2D NSs. There are also many factors to be considered in an ink formulation to ensure stable ink jetting, printing resolution, and printing pattern quality. Two different ink formulation approaches have been reported: direct ink formulation and solvent exchange ink formulation. As the names indicate, the direct ink formulation is to directly utilize the colloidal dispersion of the exfoliated 2D NSs as an ink, while in the solvent exchange formulation, the exfoliation medium is exchanged with new solvents to make the ink more suitable for printing. This section discusses the ink formulation of 2D materials, covering the exfoliation methods and two ink formulation approaches with representative works.

### 2.1. Exfoliation of Bulk 2D Materials into 2D Nanosheets

A wide range of exfoliation methods has been developed for the scalable production of 2D materials [26]. Among these, LPE is the most suitable for preparing a 2D NS dispersion for printing ink. In LPE, cavitation induced by ultrasonic-wave or high shear stress by a shear rotor is the driving force to delaminate bulk-layered materials into mono- and few-layer NSs with higher exfoliation yield [27]. Although shear stress created by a shear rotor is more efficient in improving the exfoliation yield and scalable production, 2D NSs exfoliated by cavitation are generally employed for ink formulation. This may be attributed to the operational simplicity and applicability of an ultrasonic bath or probe sonic in ordinary laboratories. Additionally, the ink for inkjet printing permits a low exfoliation yield because it requires relatively low viscosity and low concentration, compared to other printing methods such as screen printing [28,29].

It is generally preferred to use wider and thinner 2D NSs for inkjet printing. Key exfoliation conditions in ultrasonics include sonication power and treatment time. The selection of the solvent and additives is also a major determinant for yielding wider and thinner 2D NSs. Different 2D materials demand different optimal exfoliation conditions. All these complexities lead researchers to conduct exfoliation in various conditions, mostly relying on empirical studies. Table 1 summarizes the exfoliation conditions and their corresponding results represented by the dimension of exfoliated 2D NSs. A solvent of N-methyl-2-pyrrolidone (NMP) and dimethylformamide (DMF) are common exfoliation solvents regardless of the type of 2D materials. The surface tension of organic solvents and their Hansen solubility parameters (HSPs) define whether there are intermolecular interactions between the 2D materials and the solvents [30,31]. High boiling point organic solvents such as NMP and DMF have HSPs and surface tension optimized for exfoliation, and thus these solvents can efficiently produce 2D NSs without additives [16]. All of the NMP-exfoliated 2D NSs present the thickness within the few-layer range (less than ten layers) without the aid of additives (Table 1). However, the high boiling point solvents induce NS agglomeration on the substrate after printing and take a long time for evaporation, making the ink unfeasible for inkjet printing [23]. In an effort to overcome the drawbacks of high-boiling point solvents, alcohol solvents with a low boiling point are frequently used for exfoliation. Although the alcohol solvents are more affordable for printing and drying, they show the mismatch of surface tension and HSPs, causing inferior exfoliation efficiency and low dispersion stability. Yao. et al. recently reported that adding water to ethanol helped alleviate the mismatch issue and successfully obtained MoS_2_ NSs with a thickness range of 1.2~8.5 nm [32]. Alternatively, various surfactants can be added to exfoliation solvents to induce an electrostatic or steric hindrance that consequently improves the exfoliation efficiency and dispersion stability. Surfactants used for exfoliation are divided into ionic and non-ionic surfactants. Sodium cholate (SC), carboxymethylcellulose (CMC), and sodium deoxycholate (SDC) are representative ionic surfactants [33,34]. The ionic surfactants interact with the 2D material in water to balance the vdW forces in the layer to aid the exfoliation and prevent re-agglomeration of the exfoliated 2D material. However, these ionic surfactants remain after printing and cause the degradation of printed 2D NSs, requiring additional processes for removing the surfactants. Typical non-ionic surfactants include polymers such as ethyl cellulose (EC) and polyvinylpyrrolidone (PVP). The polymers are attached to 2D materials to provide a physical separation between the NS layers and enhance the dispersion stability [35,36,37,38]. In particular, EC, used in the coatings industry for decades, is mildly sonicated in exfoliation solvents to facilitate the deep insertion of EC molecules into the 2D materials’ layers, preventing the aggregation of 2D materials during exfoliation [39]. In addition to the improved ink dispersibility, it also improves the ink-jetting stability of low-boiling solvents with low viscosity that suffer from unstable ink jetting (see more details in Section 3.2). The polymer addition increases the viscosity of the ink, allowing stable jetting. Thanks to these merits, cases of using non-ionic surfactants are more prevalent than ionic surfactants [40,41].

Ultrasonic equipment that creates cavitation can also affect exfoliation performance, particularly the dimension of 2D NSs. A bath sonicator has an ultrasonic transducer at the bottom of a bath, such that the ultrasonic waves need to transfer through water in the bath. On the other hand, a probe sonicator has a relatively short transfer length of ultrasonic waves, with the result that cavitation is performed more vigorously in a probe sonicator than the bath counterpart. That is why a probe sonicator usually takes a much shorter exfoliation time than a bath sonicator. As shown in Table 1, graphene was produced following 72 h exfoliation in a bath sonicator, much longer than the 1.5 h in a probe sonicator. However, such high cavitation with a probe sonicator usually produces 2D NSs with a small lateral size, less than 100 nm in diameter, and a relatively wider lateral size range. A bath sonicator can produce 2D NSs with a larger size in a narrow size distribution, but excessive sonication treatment may create significant defects on 2D NSs [42]. Concerning the optimal exfoliation conditions of 2D materials, it is currently difficult to make a concluding mark because exfoliation conditions reported are different in each study. Therefore, more systematic and comprehensive exfoliation experiments are urgent to enlighten the optimal exfoliation conditions for ink formulation.

### 2.2. Directing Ink Formulation

**Figure 1:**
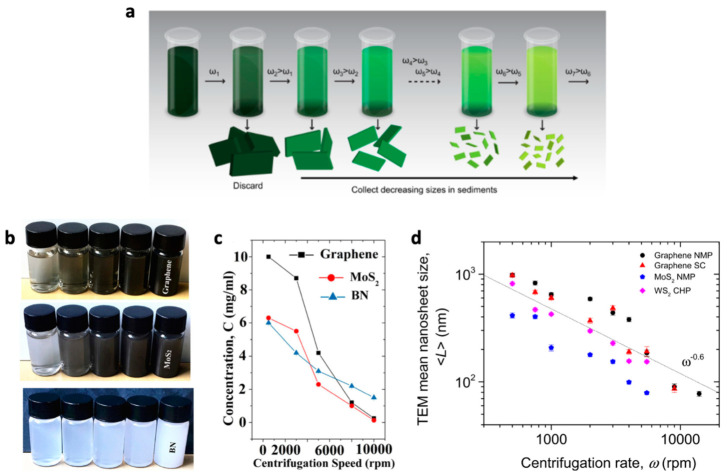
(**a**) Schematic of the size selection by liquid cascade centrifugation. Reprinted with permission from Ref. [52]. Copyright 2016 MyJoVE. (**b**) Photographs of aqueous dispersions of graphene, MoS_2_, and BN with different concentrations after centrifugation at 500–10,000 rpm for 30 min; (**c**) Lambert–Beer plots for graphene, MoS_2_, and BN; dependence of 2D material concentration on centrifugation speed for the supernatant. Reprinted with permission from Ref. [51]. Copyright 2018, IOP Publishing. (**d**) Mean nanosheet length (*L*), versus centrifugation rate (ω). Reprinted with permission from Ref. [50]. Copyright 2013 IOP Publishing.

The dispersion of exfoliated 2D NSs can be directly transferred to the ink formulation step. This direct ink formulation approach was widely adopted at an early stage of 2D NS-inkjet printing. After exfoliation, thin-layered 2D NSs coexist with unexfoliated thick flakes in the dispersion. The remaining unexfoliated contents are removed by centrifugation, leaving a supernatant of thin-layered 2D NSs to proceed to inkjet printing. Centrifugation is a representative technique for classifying 2D materials into various lateral sizes and thicknesses [15]. During centrifugation, 2D material flakes are precipitated at different RPMs, depending on their dimensions [50]. Larger or thicker flakes tend to settle because of their high mass ratio. Since the precipitation of 2D flakes is closely related to the centrifugation RPMs, the stepwise increase in centrifugation rate enables the fine size classification of 2D flake sediments (Figure 1a). The size selection by centrifugation also tailors the concentrations of 2D NSs in the dispersion: the higher the RPM of centrifugation, the lower the concentration of 2D NSs with smaller and thinner dimensions. Ding. et al. observed that the concentration of graphene, MoS_2_, and BN NSs decreased at a higher centrifugation rate where thicker and larger flakes likely precipitated at increased centrifugation rate (Figure 1b,c) [51]. A statistical TEM analysis confirms the inverse-linear relationship between 2D NS size and centrifugation rate (Figure 1d) [50]. Based on these results, it is essential to find an appropriate centrifugation rate that satisfies both the concentration and dimension of 2D NSs for printing ink. It is particularly critical for the direct ink formulation approach because the supernatant obtained after centrifugation is directly used as printing ink.

**Figure 2:**
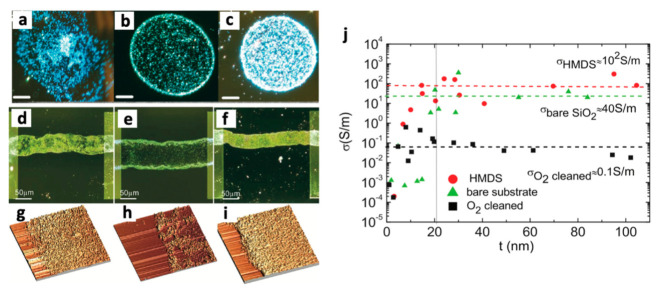
Dark-field optical micrograph of inkjet-printed drops on (**a**) plasma-cleaned, (**b**) pristine, and (**c**) HMDS-treated substrate. Scale is 20 μm. Optical micrograph of inkjet-printed stripes on (**d**) pristine, (**e**) O_2_-treated and (**f**) HMDS-treated substrates. AFM images of (**d**–**i**), respectively. (**j**) Conductivity (σ) as a function of thickness for HMDS-coated, O_2_-plasma-treated and pristine substrates. Reprinted with permission from Ref. [45]. Copyright 2012 American Chemical Society.

As one of the pioneering works in direct ink formulation, Torrisi. et al. exfoliated graphite flakes in NMP for 9 h using a sonic bath, followed by centrifugation at 10,000 rpm for 1 h to remove large flakes >1 μm in lateral size [45]. Given the optimal exfoliation and centrifugation conditions, they prepared graphene ink primarily consisting of single, bilayer, and few-layer graphene with a lateral size of 300~1000 nm. As mentioned previously, NMP is not a suitable ink solvent, such that ethylene glycol was added to increase the viscosity of the ink. Modifying the NMP-based graphene ink with the additive successfully led to the formation of stable ink jetting but failed to form a uniform pattern due to the mismatch between the surface tension of the ink and substrate. To address the surface tension issue, the authors pretreated the substrate with hexamethyldisilazane (HMDS) and improved the printing pattern quality. Nonetheless, the NMP-based graphene ink could not pattern a fine straight line of around 100 μm on the HMDS-treated substrate. The printed graphene pattern reached a maximum electrical conductivity of ≈10^2^ S/m (Figure 2j), which was much lower than that of a typical graphene pattern recently developed (≈30^3^ S/m) [53]. This study proves that the proper selection of an ink solvent plays a critical role in determining the quality of a printing pattern and the resulting electrical conductivity. However, the authors demonstrated the potential of large-area fabrication of graphene devices by pioneering graphene ink formulation early in this research field.

**Figure 3:**
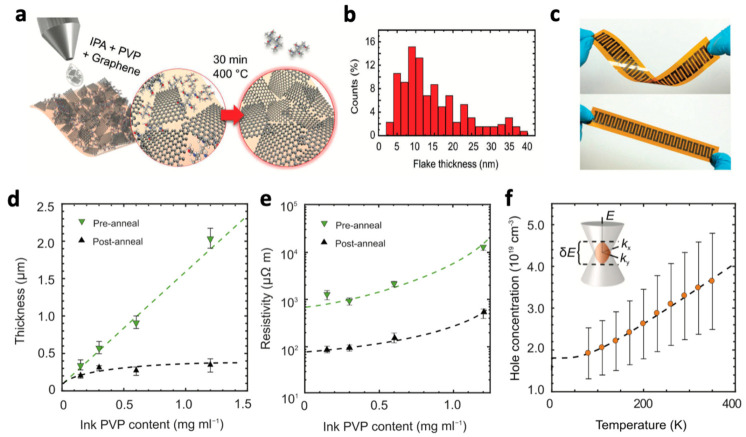
Deposition and characterization of the graphene thin films: (**a**) deposition and annealing scheme of the exfoliated few-layer graphene flakes suspended in an IPA/PVP solution; (**b**) AFM thickness distribution of graphene flakes after annealing. (**c**) Photographs of an inkjet-printed device consisting of 20 silver and graphene legs bent (above) and as is (below); thermoelectric and transport characterization of graphene films: (**d**) thickness, (**e**) resistivity as a function of ink PVP concentration pre- and post-annealing; (**f**) post-annealing charge concentration with band overlap energy δE = 28.1 ± 2.3 meV as illustrated in the inset for two parabolic bands. Reprinted with permission from Ref. [44], Copyright 2018 John Wiley and Sons.

An ink solution prepared by dissolving PVP in isopropyl alcohol (IPA), a low boiling point alcohol solvent, could be a promising approach to overcome the problems arising from NMP. Juntunen et al. exfoliated bulk graphite flakes dispersed in IPA/PVP mixture using a bath sonicator for 12 h [44]. PVP in the dispersion prevented the graphene NSs from aggregation and settlement, yielding a homogeneous graphene dispersion (Figure 3a). Fifty percent of the graphene NSs had a thickness of < 10 nm, and the average lateral size was around 200 nm (Figure 3b). The supernatant was stable and met the demands of surface tension and viscosity, and thus it could be directly used as an ink without the aid of any additives. The IPA/PVP-based graphene ink was printed on a flexible substrate for thermoelectric applications (Figure 3c). The printed graphene film showed excellent thermoelectric properties and even retained the functions against mechanical deformation tests for 10,000 bending cycles. However, the removal of PVP after printing was necessary for the excellent thermoelectric properties, implying that a trade-off between optimal ink stability and device performance dictated by the PVP concentration exists. Therefore, systematic experiments concerning the effects of PVP on ink stability and device performance should be carried out to find an optimal trade-off (Figure 3e,f).

Besides the graphene ink, MoS_2_ ink was also prepared by the direct ink formulation method. Yao. et al. selected a mixture of ethanol and water solvent as an exfoliation medium to avoid using NMP but suffered from a low MoS_2_ concentration of ~0.3 mg/mL, insufficient for inkjet printing [32]. By grinding bulk MoS_2_ flakes, followed by exfoliation in the solvent, the authors prepared a high concentration of MoS_2_ ink (26.7 ± 0.7 mg/mL). Glycerol was added to the ink to meet the viscosity and surface tension for its stable jetting. Although the combined exfoliation strategy yielded a relatively small lateral size, ranging from 20 to 60 nm, a printed MoS_2_ sensing device was fabricated, capable of detecting NH_3_ at several ppm levels for NH_3_. This study offers a promising way for how to increase the concentration of 2D materials, as a solvent bringing low exfoliation efficiency is used as an exfoliation medium.

The direct ink formulation can simplify the process by sharing the same solvent for the exfoliation and printing. However, it is likely a challenge to achieve an appropriate balance between exfoliation efficiency and printing stability. In addition, pre-or post-treatment has been occasionally required to enhance the performance of both the printing and the resulting devices.

### 2.3. Solvent Exchange Ink Formulation

**Figure 4:**
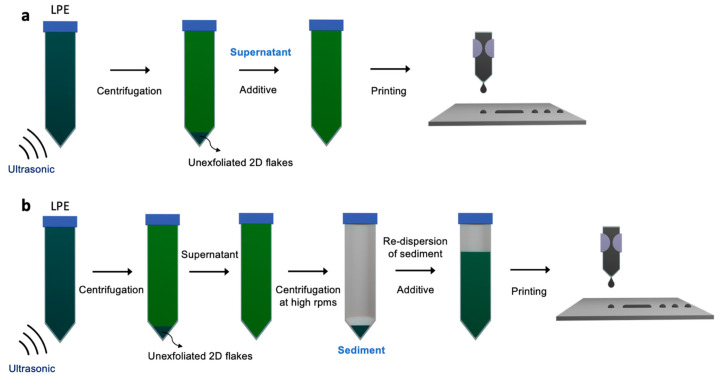
Schematic of ink formulation: (**a**) direct ink formulation and (**b**) solvent exchange ink formulation.

Solvent exchange ink formulation separates the exfoliation process and the ink formulation process (Figure 4a). This implies that the whole ink formulation procedures become more time-consuming and complicated than the direct ink formulation in which the exfoliation and ink formulation can proceed in a continuous process. The main advantages of a solvent exchange formulation are to employ high-boiling point solvents such as NMP as an exfoliation medium to ensure the exfoliation efficiency and to control the ink concentration to meet an adequate printing condition, regardless of the exfoliation yield. A general strategy of solvent exchange ink formulation is as follows (Figure 4). The 2D materials are exfoliated in a high-boiling point solvent. Then, the exfoliated 2D NSs are collected by centrifugation at high RPMs. Lastly, the collected 2D NSs are re-dispersed in new inkjet-friendly solvents.

**Figure 5:**
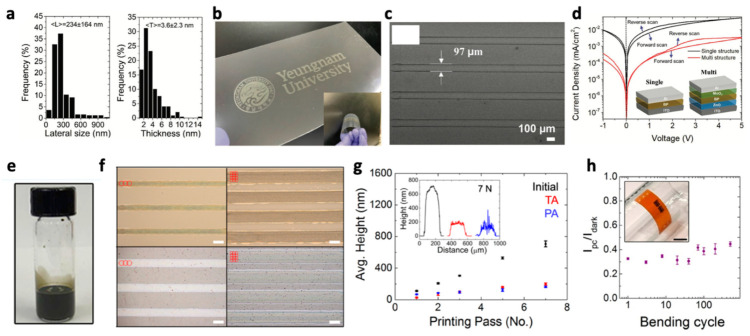
(**a**) Statistical dimensional analysis of exfoliated BP NSs; characterizations of the printed BP films upon printing repetition (N): (**b**) patterned BP film on PET substrate (100 mm × 40 mm); (**c**) SEM image of printing line at droplet spacing of 25 µm and 5 N; (**d**) diode characteristics of the single- and multi-structured BP film. Reprinted with permission from Ref. [22]. Copyright 2021 John Wiley and Sons. (**e**) Photograph of the MoS_2_/EC ink; (**f**) inkjet-printed MoS_2_/EC lines on glass (top) and polyimide (bottom). The width of the printed lines can be tuned with the number of rows of droplets per line, as indicated by the red circles. The scale bar is 100 μm; (**g**) average height measured by profilometry from the as-printed (initial), thermally annealed (TA), and photonically annealed (PA) MoS_2_ /EC lines. Inset: height profiles from the initial (black), TA (red, 177.65 ± 14.52 nm), and PA (blue, 124.68 ± 49.63 nm) lines after 7 printing passes, illustrating larger standard deviation in thickness and morphological roughness after PA; (**h**) bending test over 500 cycles showing invariant sensitivity. Inset: photograph of the flexible MoS_2_-Gr device. The scale bar is 3 mm. Reprinted with permission from Ref. [49]. Copyright 2019 American Chemical Society.

For instance, Jun. et al. exfoliated bulk BP flakes in NMP using a sonic probe. NMP was removed by a solvent exchange process, and the exfoliated BP NSs were replenished with 2-methoxyethanol (2-ME) solvent, an inkjet-friendly solvent [22]. While exchanging the solvent, the exfoliated BP NSs did not aggregate and remained in stable colloids with the average lateral size and thickness of 234 nm and 3.6 nm, respectively (Figure 5a). Interestingly, the authors claimed that 2-ME-based BP NSs ink showed a very stable ink jetting, free of additives and enabling a line pattern of 97 μm in width as well as complex printing features (Figure 5b,c). They also developed the printed BP pattern into a multi-inverse structure diode to demonstrate its potential for electronic devices (Figure 5d).

Seo. et al. exfoliated bulk MoS_2_ flakes in a mixture of ethanol and EC using a sonic bath to produce MoS_2_ NSs with a thickness of <6 nm and a lateral size of <100 nm. Then, MoS_2_ NSs were re-dispersed in cyclohexanone/terpineol (C/T) mixture by a solvent exchange process (Figure 5e) [49]. The C/T solvent has been widely used to improve the dispersibility and printing stability of 2D NSs [40,43,54]. While cyclohexanone provides a stable dispersion of MoS_2_ NSs, terpineol plays multiplying effects in improving the surface tension and viscosity for durable printing. The authors determined an optimal ink concentration by exploring a wide range of ink concentrations and their impact on printing. The printability of the ink was demonstrated by a successful line patterning of 100 μm in width, along with a proportional increase of the thickness over the printing pass, ~100 nm increment at each pass (Figure 5f,g). The authors also investigated the annealing effects on the thickness reduction and found that thermal- and photo-annealing were similar in reducing the thickness. Printed MoS_2_ patterns were fabricated for a photodetector on flexible substrates that exhibited stable photosensitivity even against the bending test of 500 cycles (Figure 5h).

With the advantages of the solvent exchange process brought by separating exfoliation and ink formulation, the efficient ink design is affordable to meet the strict requirements of inkjet printing ink. Therefore, this approach has been increasingly adopted to formulate various 2D-based inks in diversified printing research fields.

## 3. Inkjet Printing of 2D Ink

The previous section dealt with the ink formulation process. The following section discusses some critical parameters determining the quality of printing patterns and the performance of the corresponding devices. Primarily, four essential metrics, including the Ohnesorge number, coffee ring effect, droplet spacing, and percolation network, are addressed in each subsection, exemplifying some representative works.

### 3.1. Mechanism of Ink Jet Printing 

**Figure 6:**
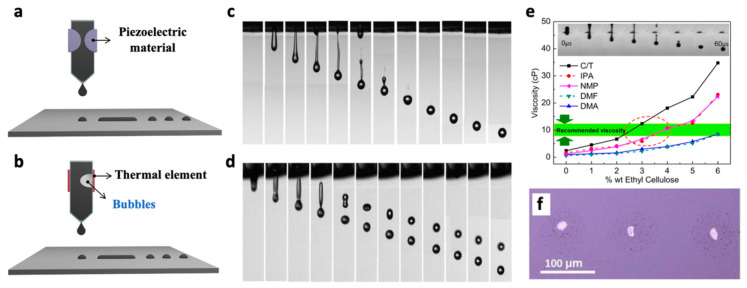
Schematics of DoD inkjet printing with (**a**) piezoelectric and (**b**) thermal head. Representative photo sequence of drop formation for fluids with (**c**) Z = 4.08 and (**d**) Z = 13.68 at a constant driving voltage of 25 V. Reprinted with permission from Ref. [56]. Copyright 2009 American Chemical Society. (**e**) Graph showing the change in viscosity of the five solvents with the addition of EC and area of recommended viscosity values for inkjet printing. Inset shows successful drop formation and ejection from the printer nozzle with minimal satellite droplets. Reprinted with permission from Ref. [41]. Copyright 2016 IOP Publishing. (**f**) Graphene line obtained with a water based ink with Z ≈ 20 and excess surfactant which aggregates in the centre of the dots. Reprinted with permission from Ref. [7]. Copyright 2017 Springer Nature.

Inkjet printing can be divided into two categories based on operating mechanism: continuous and drop-on-demand (DOD) (Figure 6a,b) [16]. In continuous printing, a continuous ink-jetting stream is ejected from a nozzle but is immediately turned into a discontinuous phase by the stream’s surface tension, called Plateau–Rayleigh instability [55]. Although continuous printing allows high-speed operation, its inherent complexities arising from strict operational requirements limit its utilization to specific applications such as label patterning. In contrast, DOD is a process in which ink droplets are ejected only on demand via a piezoelectric or thermal response. In the piezoelectric one, a voltage pulse stimulant induces the deformation of the piezoelectric material, leading to the formation and release of ink droplets. In the thermal inkjet process, the ink quickly heats up, creating air bubbles for the release of ink droplets. A DOD inkjet printing has been the primary technique due to the merits of precisely controlling the droplet size. Additionally, while the thermal DOD process generally requires volatile solvents such as water or alcohol, the piezoelectric one can afford low volatile solvents because its operation relies on the fluctuation of the piezoelectric material. The precise control in discharge rate and size of a droplet is also available by simply tuning the operating voltage. Four critical metrics that DOD printer users generally face during 2D NS-inkjet printing are discussed in the following sections.

### 3.2. Ohnesorge Number

To accomplish a stable jetting discharge in a DOD inkjet printing, several vital parameters should be considered. The ink’s surface tension and viscosity play a critical role in determining the droplet size and behavior. The nozzle size of an inkjet cartridge also has an impact on the proper ejection of ink droplets. If these parameters are not correctly adjusted, ink jetting may be impossible or satellite droplets may be generated [56]. Because satellite droplets are likely to land on untargeted areas of the substrate, they should be suppressed. The Reynolds number and Weber number can be the metrics to characterize droplet dynamics by correlating all these parameters [57]. The inverse Ohnesorge number, derived by integrating the Reynolds number and the Weber number, becomes more relevant to predict droplet dynamics [58].

(1) $$Z=\frac{1}{Oh}=\frac{\sqrt{\gamma \rho a}}{\eta }$$

where γ, ρ, and η denote the surface tension (mN m^−1^), density (g cm^−3^) and viscosity (mPa s) of an ink, respectively, and a is the diameter of a jetting nozzle. It has been known that if the ink has a Z value of 1 < Z < 14, stable droplets can be steadily formed. At high viscosity (Z < 1), the ink cannot discharge from the nozzle, and at low viscosity (Z > 14), it likely forms satellite droplets. Figure 6c shows a stable droplet jet at Z = 4.08, and a satellite droplet at Z = 17.32 (Figure 6d). Therefore, the Ohnesorge number offers guidance on the parameter range for steady ink jetting as well as the prediction of printing pattern quality of DOD inkjet printing [56,58]. Among the parameters of the Ohnesorge number, the viscosity is the most influential in the ink rheology, and by adding polymer surfactants such as EC, it can be easily tailored. Michel. et al. reported that the printable viscosity range was achieved after adding EC to various solvents such as C/T, IPA, NMP, DMF, and *N*,*N*-Dimethylacetamide (DMA), demonstrating EC’s versatile role in adjusting the viscosity for printing [41]. This surfactant also disrupts agglomeration and sedimentation of 2D NSs in ink, preventing nozzle clogging. In this context, an optimal surfactant amount should be determined in each printing trial because its overuse has a detrimental effect on the printing. For example, the polymer surfactant inhibits the conductivity of printed graphene patterns. Although high-temperature annealing enables the removal of the surfactants, their residue is still present in the printing pattern. Moreover, some 2D materials including BP NSs quickly degrade via oxidation in atmospheric conditions [20]. Because high-temperature annealing accelerates oxidation, surfactant addition should be avoided during BP ink formulation. Excessive use of the surfactant also causes aggregation of 2D NSs on the substrate after printing. In Figure 6f, a printed graphene line was initially intended using a water-based graphene ink modified with surfactant. The remained surfactant interrupted the uniform distribution of graphene and instead led to graphene’s aggregation to form an array of irregular dot patterns [7]. These results indicate that polymer surfactants should be carefully manipulated, taking into account the type of 2D materials, applied substrate, and target patterning.

### 3.3. Coffee Ring Effect

**Figure 7:**
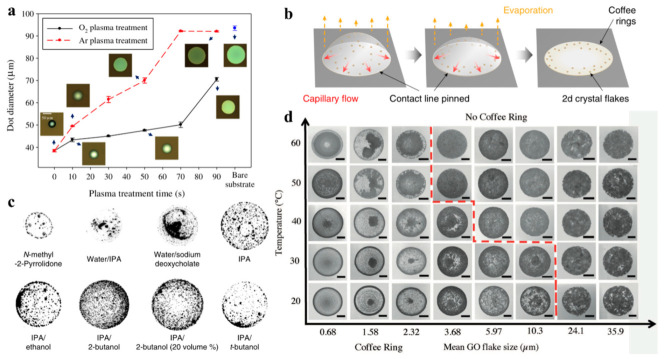
(**a**) Variation in inkjet-printed dot diameter as a function of O_2_ or Ar plasma treatment time. Reprinted with permission from Ref. [62]. Copyright 2013 Elsevier. (**b**) Schematic drying process showing CRE formation; (**c**) inverted optical micrographs of dried inkjet-printed droplets on clean glass: common solution-processed 2D crystal dispersions. Formulated inks via solvent exchange in IPA or binary solvents of IPA/ethanol (10 volume %), IPA/2-butanol (10 and 20 volume %), and IPA/t-butanol (10 volume %). Reprinted with permission from Ref. [23], Copyright 2020 American Association for the Advancement of Science. (**d**) SEM image map for printed droplets in rows with increasing substrate temperature and in columns from left to right with increasing mean GO flake size. The dotted line indicates the parameter space where the coffee ring is fully suppressed. All scale bars are 100 µm. Reprinted with permission from Ref. [42]. Copyright 2017 John Wiley and Sons.

Once an ink droplet is stably jetted from a nozzle, it settles on the substrate and proceeds to spreading and drying. The spreading of ink over a substrate is defined by wetting, which can be explained by Young’s equation:

γ_sv_ = γ_si_ + γ_iv_ cos θ (2)

where γ_sv_, γ_iv_ and γ_si_ represent the respective interfacial tensions between the substrate (s), the vapor (v), and the ink (i), and θ is the formed contact angle. The spreading and drying rate of the droplet depends on the surface energy difference between the droplet and substrate, depicted by the wettability. It is generally known that adequate wettability can be achieved when the surface tension of the ink is 7–10 mN m^−1^ lower than the substrate’s surface energy [16]. There are many practical substrates used in printing industries, including Si/SiO_2_ and glass. Recently, wearable substrates such as polyimide (PI), polyethylene terephthalate (PET), Kapton, cotton, and paper are also increasingly applied [17,29,32,37,59,60]. Because these substrates have a different intrinsic surface energy, the surface tension of the ink should be properly engineered to ensure the appropriate wettability. However, as discussed previously, an ink solvent is concerned with the exfoliation efficiency, ink stability, and steady ink jetting, such that simply engineering ink toward the proper wettability is not straightforward. A more feasible way to achieve good wettability is through the surface treatment of the substrate. The most common surface treatment utilizes O_2_ plasma or UV/O_3_, improving the wettability for lower surface energy [61,62]. These surface treatments allow large-area surface modification in a simple manner, along with time-dependent wettability control by forming hydroxyl groups on the surface of the substrate. Park. et al. revealed that O_2_ plasma treatment time changed the diameter of a printed silver dot (Figure 7a) [62]. The diameter of a silver dot reached up to 90 μm from 40 μm after plasma treatment for 90 s. Functionalizing the surface with HMDS molecules can lower the wettability. During HMDS treatment, a hydroxyl group (−OH) is replaced with a silane group (−Si(CH_3_)_3_), resulting in higher surface energy. This treatment is frequently beneficial, particularly for the direct printing of an NMP-based 2D NS ink [45]. These indicate that adjusting the surface wettability into higher or lower one exclusively depends on the type of applied ink. Nonetheless, fine control of the surface wettability through the treatment offers the potential to diversify ink solvents, which would expand inkjet printing toward various 2D materials.

Following decent contact of an ink droplet to the substrate, an ink droplet diffuses and evaporates over the substrate simultaneously. Due to the low ink viscosity, printed 2D NSs tend to concentrate on the pattern edges upon evaporation, leading to the formation of a coffee ring and subsequently a concave pattern (Figure 7b) [23]. The coffee ring is triggered as the solvent evaporates more rapidly at the edge of the droplet than the central area, creating a contact line pinned at the edge. The capillary flow also promotes the spreading of the solvent toward the edge, accelerating the coffee ring formation (Figure 7b). A non-uniform pattern degrades device performances significantly and disrupts the reproducible and scalable fabrication of printing patterns. Therefore, effort has been made to suppress coffee ring formation. Representative of this, Hu. Et al. harnessed the Marangoni effect to relieve the coffee ring effect [23]. The Marangoni flow involves the mass transfer driven by a surface tension gradient of solvents in a solvent mixture, such that adequately tuning a surface tension gradient by mixing two solvents could manipulate the flow dynamics of the solvents and therefore the particle position on the substrate as well. Inspired by this mechanism, the authors prepared a series of mixtures comprising two solvents with different surface tensions and investigated the coffee ring formation of the mixtures (Figure 7c). They found that a mixture of IPA and 2-butanol is the best formulation to suppress the coffee ring formation. In this system, a uniform 2D NS pattern was printed, although different 2D NSs demanded different combinations of solvents. 

He. et al. claimed that tailoring the size of 2D NSs and the substrate temperature could address the coffee ring issue [42]. The comparison of printed graphene oxide (GO) morphology acquired from various experimental conditions suggested elevated temperature is generally beneficial for weakening the coffee ring effect (Figure 7d). When it comes to the size effect of GO, using larger GO was preferred to suppress the coffee ring formation, and this size effect became more pronounced at elevated temperatures.

Recently, the ink concentration was reported to be an effective parameter in controlling the coffee ring effect. Jun. et al. varied the concentration of BP NSs in ink in a wide range and found that a concentration over 1 mg/mL effectively weakened the coffee ring effect [22]. However, it is unclear whether the concentration is solely an influential factor for coffee ring inhibition. It is also uncertain that this concentration effect still holds for 2D NSs other than BP NSs.

### 3.4. Droplet Spacing

**Figure 8:**
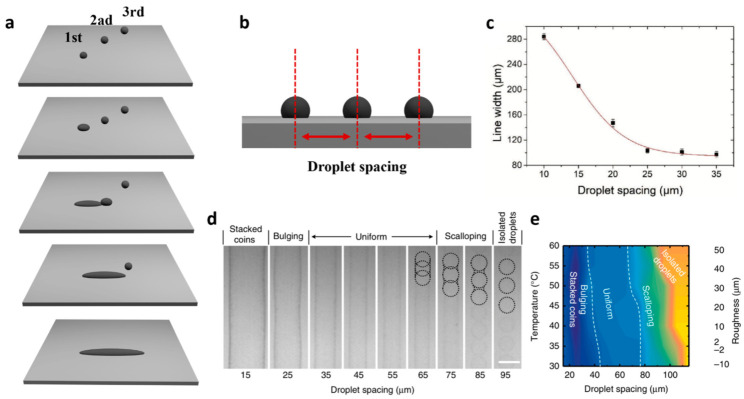
(**a**) Schematic of 2D material patterning process and (**b**) droplet spacing. Characterizations of the printed BP films upon printing repetition (N): (**c**) printing line width variation with respect to droplet spacing. Reprinted with permission from Ref. [22]. Copyright 2021 John Wiley and Sons. (**d**) Optimization of BP printing conditions. a BP printed on Si/SiO_2_ at 60 °C showing the effect of droplet spacing on line morphology, photos taken by printer fiducial camera, scale bar, 100 μm; (**e**) effect of droplet spacing and printing temperature on the roughness along line edges, the roughness from uniform to stacked coins is defined as negative. Reprinted with permission from Ref. [20]. Copyright 2017 Springer Nature.

Vital parameters involving DOD inkjet printing are substrate temperature and droplet spacing that define the morphology of printed patterns. Figure 8a is a schematic diagram to show how ink droplets complete a line pattern. Ink droplets ejected from a nozzle sequentially combine with the neighboring one to constitute a line pattern. The droplet spacing refers to the distance between ink droplets sitting on the substrate (Figure 8b). Since droplet spacing significantly influences the morphology of printing patterns such as line width, thickness, and uniformity, it should be explored to find the optimum [20]. Figure 8c exhibits the line width variation of printed BP NSs with respect to droplet spacing [22]. At a droplet spacing of less than 20 µm, droplets began to overlap considerably and eventually formed a broad line up to 280 μm at a 10 µm droplet spacing. On the other hand, when droplet spacing was 25 μm, the printing line width was reduced almost one-third to 97 μm and remained consistent beyond that. This reflects the existence of a droplet spacing threshold that limits the line width control. Hu. et al. studied the correlating effects of droplet spacing and substrate temperature on the morphology of printed BP patterns [20]. A narrow droplet spacing caused the droplets to overlap significantly, resulting in a broader line (stacked coins in Figure 8d). A drop spacing from 75 μm through 85 μm led to the formation of scalloped lines due to insufficient ink to merge, and a drop spacing beyond that resulted in isolated droplets that were too distant to merge. In this experiment, an optimal droplet spacing was revealed to range from 35 μm to 65 μm within which a uniform line pattern was printed. Because the substrate temperature affects the evaporation rate of the solvent and behaviors of printed 2D NSs, printing morphology could vary with the temperature applied. As shown in Figure 8e, both the morphology uniformity and optimal droplet spacing range were changed, compared to those at a constant substrate temperature (Figure 6d). 

### 3.5. Percolation Network

**Figure 9:**
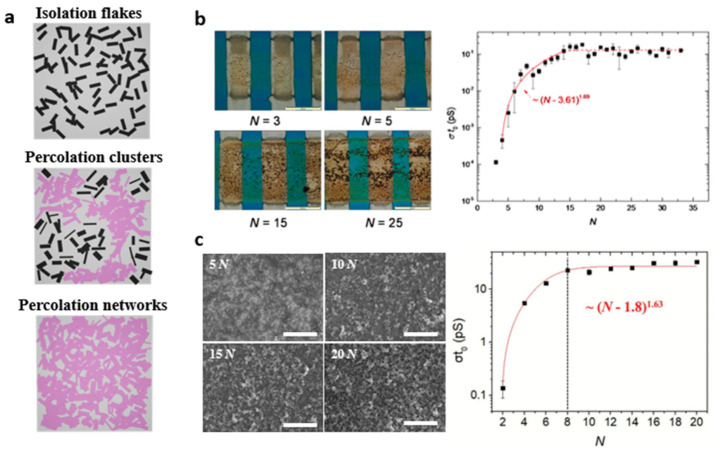
(**a**) Schematic of percolation theory: isolation flakes, percolation clusters, percolation networks. (**b**) Optical micrograph of the printed MoS_2_ transistors on Si/SiO_2_ wafers with different printing passes N; conductivity variation of the printed BP films with respect to N. Reprinted with permission from Ref. [36]. Copyright 2014 John Wiley and Sons. (**c**) SEM images of BP films printed at 5 N, 10 N, 15 N, and 20 N (Scale bar = 10 µm); conductivity variation of the printed BP films with respect to N. Reprinted with permission from Ref. [22]. Copyright 2021 John Wiley and Sons.

The percolation theory describes a state of connectivity between particles and can be divided into three phases of the state (Figure 9a). In an isolation state, particles are separated with little contact. Then, more particles participate in the connection and form into clusters, called percolation clusters. Although the percolation clusters are established through the linking of many particles, they are still insufficient to form a network structure across the entire surface. By engaging more particles with the percolation clusters, the percolation networks are completed [63,64]. Many materials, such as carbon nanotubes, graphene, and nanocomposites, have been tested to attempt to reach the percolation networks via inkjet printing [65,66]. The percolation theory can also be applied to assess the connectivity of printed 2D NSs. Particularly, the electrical conductivity of printed 2D NSs is a reflection of the percolation state. The electrical conductivity of printed 2D NSs rapidly increases at the entry of the percolation networks, so-called the percolation threshold. Figure 9b displays that printing repetition (N) could control the progress of the percolation process for printed MoS_2_ NSs. At a low N value between 3~5, MoS_2_ NSs were within isolation or percolation clusters, and thus the electrical conductivity was inferior. As printing repetition approached 15, MoS_2_ NSs were deposited in a high population, enough to fulfill the percolation threshold and permitted electrons to flow through the percolation networks. Once the percolation threshold was met, additional printing passes no longer helped to increase the electrical conductivity. It is reasoned that larger 2D NSs are pursued to establish the percolation threshold for the least number of printing passes. Larger 2D NSs are also beneficial for high electrical conductivity because electrons can flow better in a low density of grain boundaries. That is why an optimal exfoliation condition should be explored to produce larger and thinner 2D NSs during the exfoliation process. As demonstrated by the printed MoS_2_ NSs, increasing the printing passes is the most straightforward way to achieve the percolation threshold of 2D NSs. Another example belongs to printed BP NSs whose percolation network was formed by repeating the printing pass up to 8 times (Figure 9c). The morphology of printed BP NSs taken at different printing repetitions corresponded well to the electrical conductivity results. It seems that printed BP NSs featured percolation clusters at 5 N and reached the percolation network at around 10 N. Although printing repetition offers the most straightforward way to produce the percolation networks, it is achieved by compensating printing time and efficiency. Therefore, ink concentration and droplet spacing should also be tailored concurrently to accomplish the percolation network efficiently.

## 4. Application

The primary goal of this review is to account for ink formulation and printing parameters that affect printing pattern quality and printing efficiency. Several review articles have already been published to explain the applications of printed 2D NSs in detail. Therefore, this section summarizes the application trends of printed 2D NSs without addressing a specific work. As outlined in Table 2, a variety of 2D NS inks have been formulated for inkjet printing. The vast majority of printed 2D NSs belong to graphene and GO due to their unique properties and excellent processibility. The high intrinsic electrical conductivity of graphene is attractive in some research fields, including printed electrodes and microsupercapacitors. Graphene also features excellent thermal conductivity, finding applications for thermal treatment. Although GO is inferior to graphene in thermal and electrical conductivity, its excellent processibility makes GO ink formulation more feasible for various applications. In particular, GO could be reduced to recover the intrinsic properties of graphene to the extent of being applicable for chemical sensors and photodetectors. Following graphene and its derivatives, various 2D materials have emerged for inkjet printing applications, and ink formulation strategies have correspondingly diversified to accommodate them. The successful settlement of these materials in inkjet printing has led printed 2D NSs to expand their applications to the semiconductor area that graphene families are hardly used due to an almost zero band gap. Particularly, BP has a tunable band gap depending on its thickness, and thus printed BP NSs were employed for diode and photodetector. Other semiconducting 2D NSs such as MoS_2_ and WS_2_ could also share similar applications with BP. BN, an intrinsically insulating material, has been frequently printed for some applications. Since MXene appeared as a promising alternative to graphene, printed MXene has recently attracted considerable interest from many research fields. The represented applications of printed MXene include microsupercapacitors and energy storage devices, such as rechargeable batteries. The advantage of MXene over graphene is its potential for scalable production and improved processibility, although the exfoliation process of MXene generally involves harsh environments. Hybrid inks formulated by mixing 2D NSs with some foreign materials have been printed to harness either the synergetic effects or multiplying effects, consequently improving the performance of the target devices.

With a wide range of 2D NSs available for ink formulation and the scalable patterning of inkjet printing, printed 2D NSs become the center of electronics and miniaturized energy storage devices. The post-treatment process to decompose additives after printing has also advanced to fabricate printed 2D NSs for wearable electronics. The fabrication strategies for flexible and stretchable electronics have been driven by the huge demand for wearable, intelligent, and integrated electronics systems. Wearable electronic applications require functional materials and manufacturing strategies. The inkjet printing process and 2D material inks are new trends in the field of printing electronics for flexible and stretchable electronics [67,68]. Based on the enormous progress made so far, it is believed that printed 2D NSs will offer a promising platform for manufacturing next-generation wearable devices, sensors, biodevices, and energy conversion devices.

## 5. Summary and Future Prospects

This review underscores the optimizing conditions for ink formulation and printing parameters for inkjet printing of 2D NSs by compiling the strategies that have been suggested over the years. Table 3 lists the relationships to correlate the printing strategies with the vital printing parameters that determine the printing quality of 2D NSs. The exfoliation process correlates with the dimension of exfoliated 2D NSs, ink concentration, and solvent and additives. Droplet stability is primarily determined by ink concentration, solvent, and additives. The coffee ring effect and percolation network involve nearly all of the printing strategies. These relationships indicate that a printing strategy interplays with several printing parameters, such that it should be tailored interactively. For ink formulation, exfoliation of bulk 2D materials and ink stability should be first considered. Using a suitable exfoliation solvent could improve the exfoliation efficiency, but it could compromise the ink stability and ink jetting. Adding surfactants to ink solvent could improve the ink stability, but the surfactants may adversely affect the quality of printed 2D NSs. In this regard, the most efficient way for ink formulation is to employ the solvent exchange ink formulation, with which the efficient exfoliation of 2D NSs and stable ink dispersion could be achieved. 

In recent years, the applications of printed 2D NSs have extended to various research fields, including optoelectronics, photonics, sensors, and energy storage, with significant advances. However, there remain challenges to achieving the commercial viability of printed 2D NS devices. First, it is necessary to improve the exfoliation yield of 2D NSs to supply high-quality 2D NS ink at a low cost. Because the exfoliation yield by LPE is typically below 10%, other exfoliation methods should be introduced to improve the exfoliation yield. In the centrifugation process, it is difficult to precisely classify the thickness and size of 2D NSs by their weight, and many thick flakes are still dispersed in printing ink. Therefore, a methodology to precisely assort the exfoliated 2D NSs for thickness and lateral size is urgent in order to manufacture high-performing printed 2D NS devices. These challenges are in effect as long as 2D NSs-based applications are associated. Thus, numerous efforts have increasingly been devoted to resolving the issues in a 2D material research community. The ink formulations and printing parameters suggested so far are based on empirical studies. Fundamental studies should be conducted to deliver more scientific insight into the ink formulation and printing parameters. Lastly, printed 2D NSs are mainly dedicated to graphene or GO, and thus diversifying 2D NSs for inkjet printing is required to heighten the potential of printed 2D NSs. It seems that establishing the commercial viability of printed 2D NSs is a long way from now. However, once these hurdles are removed, commercial viability may be realized.

## Figures and Tables

**Table 1:** Two-dimensional materials produced by LPE.

Materials	Exfoliation				2D Materials		Ref.
	Solvent	Surfactant and Binders	Sonication	Time (h)	Thickness	Lateral Size	
Graphene	DMF	EC	Bath	40	-	100–500 nm	[43]
Graphene	IPA	PVP	Bath	12	<10 nm	200 nm	[44]
Graphene	IPA	PVP	Bath	12	<5.9 nm	196 nm	[29]
Graphene	NMP		Bath (20W)	9	Single	300 nm	[45]
Graphene	NMP	CMC	Bath	9	6 nm	121 nm	[34]
Graphene	Water	PS1 salt	Bath (300W)	72	<10 layer	400 nm	[7]
Graphene	Cyclohexanone	EC	Probe (120W)	7	< 1 nm	30–100 nm	[46]
Graphene	Ethanol	EC	Probe (50W)	1.5	<2 nm	50 nm	[40]
Graphene	NMP		Probe (120W)	7	<10 layer	35–600 nm	[17]
Graphene BN	NMP IPA		Probe (120W)	1.5	<8 nm	195 nm 450 nm	[47]
BP	NMP		Probe (120W)	1	3.6 nm	234 nm	[22]
BP	NMP		Probe	12	3.37 nm	80.46 nm	[20]
MoS_2_	DMF	EC	Bath	48	<7 nm	40–100 nm	[36]
MoS_2_	Ethanol, water	PVP	Bath	48	-	100–200 nm	[48]
MoS_2_	Ethanol, water		Bath (with grinding)	2	1.2–8.5 nm	20–60 nm	[32]
MoS_2_	Ethanol	EC	Shear mixer	2	<6nm	<100 nm	[49]

**Table 2:** Two-dimensional material inks and their printable applications.

Materials	Exfoliation		Ink Formulation		Substrate	Application	Ref.
	Solvent	Surfactant and Binders	Solvent	Surfactant and Binders			
**2D Material inks**							
Graphene	Cyclohexanone	EC	Cyclohexanone	EC	Si/SiO_2_, PI, PET	Conductive ink	[46]
Graphene	DMF		C/T	EC	Kapton, Glass	Microsupercapacitors	[43]
Graphene	DMF	EC	Terpineol/ ethanol		Kapton, Glass	Microsupercapacitors	[37]
Graphene	Ethanol	EC	C/T	EC	Si/SiO_2_	Conductive ink	[40]
Graphene	IPA	PVP	IPA	PVP	Si/SiO_2_	Thermoelectrics	[44]
Graphene	IPA	PVP	IPA	PVP	Glass	Solar cells	[29]
Graphene	NMP		NMP	Ethylene glycol	Si/SiO_2_	Conductive ink	[45]
Graphene, h-BN	NMP Water	CMC	Ethanol Water		Textile	Conductive ink	[34]
Graphene, MoS_2_	IPA, C/T, DMA, DMF, NMP	EC	NMP	EC	Si/SiO_2_, PET	Conductive ink	[41]
Graphene BN	NMP IPA		NMP IPA		PET	Capacitors	[47]
Graphene, MoS_2_	IPA, C/T, NMP		C/T	EC	PET, Pi	Photodetectors	[60]
Graphene, MoS_2_	NMP		NMP		Glass	Photodetectors	[69]
Graphene, MoS_2_	NMP		NMP		PET	Conductive ink	[17]
Graphene, WS_2_	Water	PS1 salt	Water	Triton x-100, Propylene glycol	Paper	Photodetectors	[70]
Graphene, WS_2_, MoS_2_, BN	Water	PS1 salt	Water	Triton x-100, Propylene glycol	Si/SiO_2_, PI, Quartz, PET	Photodetectors, memory	[7]
BP	NMP		2ME		Si/SiO_2_	Diode	[22]
BP	NMP, CHP, IPA		IPA	2-butanol	Si/SiO_2_, Glass, PET	Photonic device	[20]
MoS_2_	Water/IPA	PVP	Water/IPA	Propylene glycol	PI	Microsupercapacitors	[18]
MoS_2_	DMF	PVP	IPA	PVP	Si/SiO_2_	CMOS logic	[71]
MoS_2_	DMF	EC	DMF/ Terpineol/ Ethanol		Si/SiO_2_	Photodetectors	[36]
MoS_2_	Ethanol	EC	C/T		Glass, PI	Photodetectors	[49]
MoS_2_	Ethanol, water	PVP	Ethanol/water / n-propanol	Glycerol/ethylene glycol	paper	Conductive ink	[48]
MoS_2_	Ethanol, water		Ethanol/water	Glycerol	Si/SiO_2_	Gas sensor	[32]
MXene	Water		NMP		Paper	Energy Storage Devices	[72]
MXene	Water		DMSO		PET	Electromagnetic shielding	[59]
MXene	Water, NMP		Water, NMP	DMSO/DMF/ Ethanol	PET, Glass, Kapton	Microsupercapacitors	[9]
MXene	IPA		IPA		Si/SiO_2_, PET	Laser	[21]
MXene, GO	Water		Water	Triton x-100, Propylene glycol	Si/SiO_2_, PI	Microsupercapacitors	[73]
WS_2_	C/T	EC	C/T	EC	Si/SiO_2_	Photodiode	[54]
rGO	Water	PVA	Water	Glycerol/ Triton x-100	Si/SiO_2_, Cotton fabric	Wearable application	[74]
GO	Water	Ethylene glycol	Water	Ethylene glycol	Glass	H_2_O_2_ sensor	[38]
GO	Water		Water		Si/SiO_2_	Conductive ink	[42]
							
Hybrid ink							
Graphene/Ag	C/T	EC	C/T	EC	Si/SiO_2_	Conductive ink	[75]
MXene/ PEDOT:PSS	Water		Water	Ethylene glycol	PET	Microsupercapacitors	[76]
Mxene/Ag	NMP		Water	Ethylene glycol	PET, PEN	Touchless sensor	[77]
MXene/GO	Water		Water	Nafion polymer	Glass, gold foil	Hydrogen peroxide sensor	[78]

**Table 3:** Factors for 2D material printing performance and their correlation with each parameter.

Printing Metrics	Dimension of 2D NSs	Ink Concentration	Solvent and Additive	Substrate Treatment	Substrate Temperature	Droplet Spacing	Printing Repetition
Exfoliation	✓	✓	✓				
Droplet stability		✓	✓				
Coffee ring effect	✓	✓	✓	✓	✓	✓	
Percolation network	✓	✓	✓	✓	✓	✓	✓

## Data Availability

Not applicable.
